# High-resolution ultrasound vs. MR neurography in upper extremity neuropathies: exploratory analysis of perceived additional diagnostic value in routine clinical practice

**DOI:** 10.1007/s00330-026-12545-0

**Published:** 2026-04-23

**Authors:** Merle Brunnée, Olivia Foesleitner, Marietta Kirchner, Fabian Preisner, Moritz Kronlage, Tim Godel, Johann M. E. Jende, Tim Hilgenfeld, Sabine Heiland, Markus Weiler, Martin Bendszus, Daniel Schwarz

**Affiliations:** 1 Department of Neuroradiology, Medical Faculty, Heidelberg University and Heidelberg University Hospital, Heidelberg, Germany; 2 Institute of Medical Biometry, University of Heidelberg, Heidelberg, Germany; 3 Department of Neurology, Medical Faculty, Heidelberg University and University Hospital, Heidelberg, Germany

**Keywords:** Comparative imaging study, Diagnostic imaging, High-resolution ultrasound, MR neurography, Peripheral neuropathy

## Abstract

**Objectives:**

High-resolution nerve ultrasound (HRUS) and magnetic resonance neurography (MRN) are the imaging modalities of choice for evaluating peripheral neuropathies. This study aimed to identify predictors and practice-patterns of perceived additional diagnostic value in clinical routine for the two modalities in patients with suspected upper extremity neuropathies.

**Materials and methods:**

This retrospective exploratory subanalysis was conducted within a prospective, observational, single-center study of 800 patients referred for HRUS and MRN between November 2015 and February 2022 for suspected upper extremity neuropathy. Patients were included where one modality demonstrated perceived additional diagnostic value over the other, defined as the detection of clinically relevant lesions missed by the other or by providing additional significant diagnostic information. Predictors of perceived additional diagnostic value were assessed using uni- and multivariable logistic regression and a classification regression tree (CART) model.

**Results:**

Of the entire cohort (800 patients), 275 (34.4%) demonstrated perceived additional diagnostic value of one modality. MRN provided additional diagnostic value in 261 cases (94.9%) and HRUS in 14 (5.1%). Additional diagnostic value of MRN was predicted by proximal nerve lesions (proximal only: OR 5.72, 95% CI: 3.42–9.56; proximal-peripheral combined: OR 4.59, 95% CI: 2.31–9.09), multi-anatomical region-involvement (OR 1.81, 95% CI: 1.14–2.87), and multi-nerve involvement (OR 1.65, 95% CI: 1.03–2.63). HRUS added value mainly when MRN was limited by metal artifacts.

**Conclusion:**

Proximal nerve lesion location, multi-regional involvement and multi-nerve pathology were found to favor MRN. On the other hand, HRUS proved particularly beneficial in the presence of metal implants.

**Key Points:**

***Question*** *Which predictors can be identified to guide the choice of preferred imaging modality—high-resolution ultrasound or MR neurography—in suspected upper extremity peripheral neuropathies?*

***Findings*** *Proximal nerve lesion location, multi-regional involvement and multi-nerve pathology were found to favor MR neurography, while HRUS proved beneficial in the presence of metal implants.*

***Clinical relevance*** *Our results provide an exploratory imaging rationale for patient-tailored diagnostic strategies in peripheral neuropathy care.*

**Graphical Abstract:**

**Figure:**
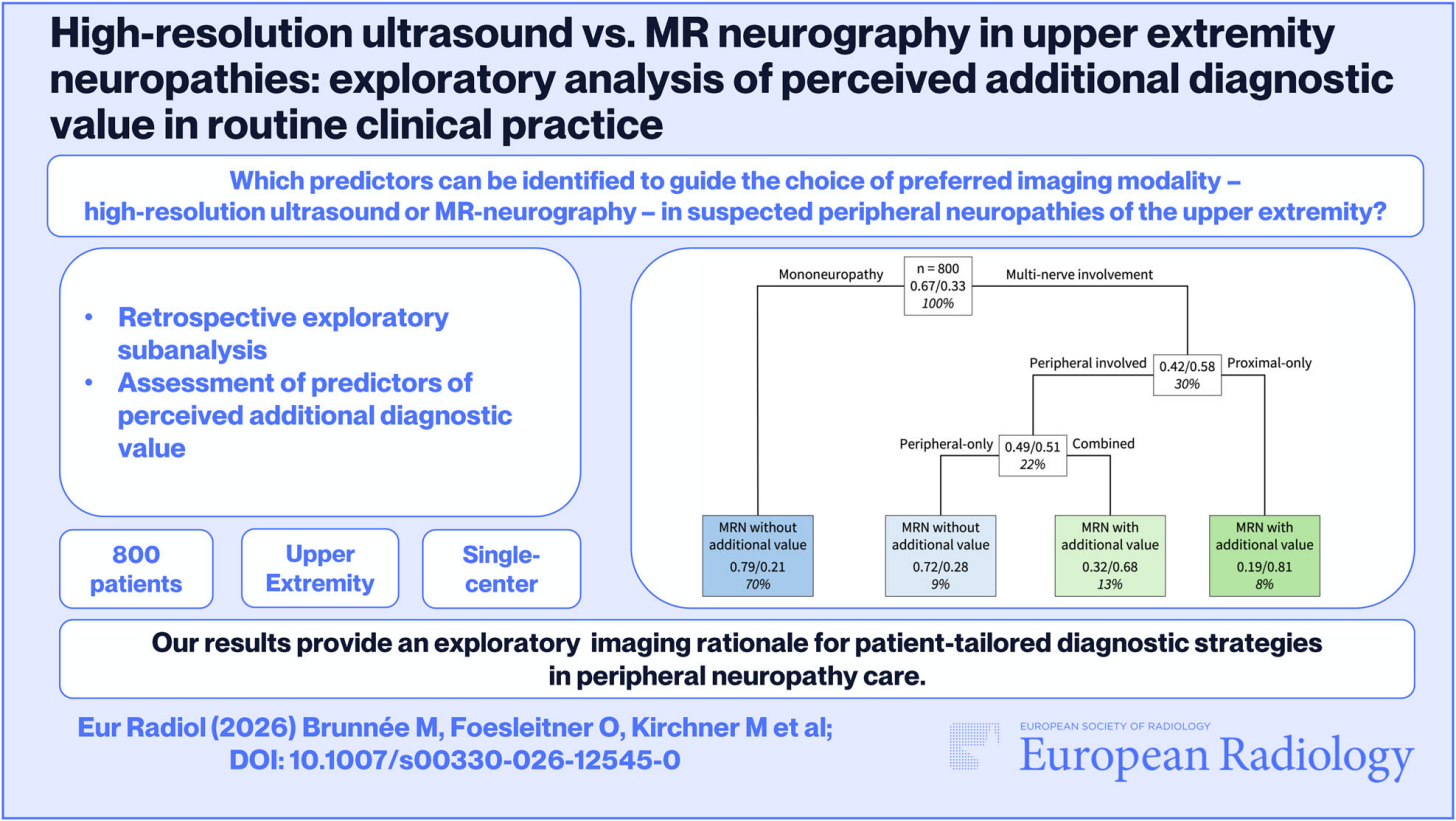

## Introduction

Peripheral neuropathies of the upper extremity are common and often disabling conditions of diverse etiology, including trauma, entrapment, inflammation, and neoplastic or systemic disease [1, 2]. Accurate diagnosis relies on a combination of clinical evaluation, electrophysiological testing, and, increasingly, advanced imaging to localize and characterize nerve pathology [3, 4]. While clinical and neurophysiological examinations remain the foundation of peripheral nerve assessment, these approaches have several limitations and often lack sensitivity in determining lesion extent or precise anatomical localization—particularly in complex or proximal nerve injuries [5, 6].

High-resolution nerve ultrasound (HRUS) has become a widely used first-line imaging tool due to its portability, real-time dynamic assessment, and cost-effectiveness [7]. It is particularly useful in superficial and peripheral mononeuropathies, where structural abnormalities such as nerve entrapments, discontinuities, or fascicular swelling can be readily visualized [8]. Magnetic resonance neurography (MRN), by contrast, offers multiplanar, high-contrast imaging of nerves throughout the upper extremity and brachial plexus, and enables the detection of deeper or more widespread pathology, including nerve edema, inflammation, and muscle denervation [4, 9, 10]. Moreover, MRN provides high-resolution anatomical findings and information on soft tissue composition by the application of different MR sequences. Several studies have demonstrated the diagnostic utility of both modalities in peripheral nerve disorders, but direct comparisons remain limited and often focus on sensitivity, specificity, accuracy, or agreement rates [11–15].

In clinical practice, the choice between HRUS and MRN as first-line imaging modality can be challenging—especially in patients with nonspecific symptoms or inconclusive electrophysiology [15, 16]. There is therefore a need to identify clinical scenarios when one modality may provide additional diagnostic value over the other. This knowledge could improve imaging workflows, diagnostic yield, and resource allocation.

Building on our previously reported single-center study of 800 consecutive patients [17], which provided important groundwork for understanding the diagnostic performance of MRN and HRUS, we conducted the present exploratory subanalysis. While the original study demonstrated that MRN had higher overall diagnostic accuracy and sensitivity, and HRUS offered higher specificity, it did not resolve the clinically crucial question under which circumstances one modality should be prioritized. The current study therefore specifically investigates cases with discordant findings between MRN and HRUS, aiming to identify key predictors and practice-patterns of perceived additional diagnostic value in clinical routine, favoring one of the two modalities.

## Methods

### Study design and participants

This study reports a retrospective exploratory subanalysis of a previously published prospective, observational, single-center cohort study [17] approved by the institutional review board, with written informed consent obtained from all participants. The study adhered to the STROBE guidelines for observational research [18], and reporting of prediction models follows the TRIPOD guidelines [19].

The total cohort comprised 800 consecutive patients with clinically suspected upper extremity neuropathy who were referred for diagnostic MRN and HRUS between November 2015 and February 2022 at our center. All patients underwent both imaging modalities, with reports generated independently for each modality. Each patient was considered a single case, even in the presence of multiple symptomatic nerves.

**Fig. 1:**
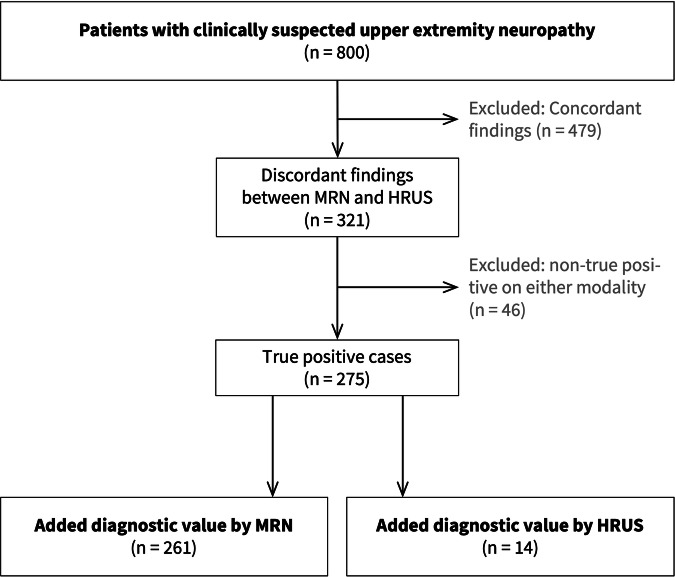
Overview of patient inclusion and exclusion steps, and final allocation to groups based on perceived additional diagnostic value of MR neurography (MRN) and high-resolution ultrasound (HRUS)

From the total study cohort (*n* = 800), we first identified all cases with diagnostically discordant findings between the two modalities excluding non-true positives (compare Fig. 1). These cases were categorized as either having perceived additional diagnostic value in the routine clinical practice of MRN or HRUS based on predefined criteria. This binary outcome variable, perceived additional diagnostic value (yes/no), was defined as either:

- a positive finding on one modality with a negative result (non-diagnostic or normal) on the other; or
- both modalities yielding positive findings, but one modality contributing further crucial diagnostic information considered clinically significant, as defined in Supplementary Table 1.

However, despite these definitions, it should be noted that this outcome variable can be subjective, is institution-specific, and not externally validated. It may therefore not be interpreted as an objective diagnostic accuracy outcome.

The final diagnosis as documented in the clinical records served as the reference standard, based on a non-algorithmic, clinician-adjudicated and therefore inherently subjective composite of clinical examination, electrodiagnostic testing, imaging, and, when available, surgical or histopathological confirmation documented in clinical records [17]. If no unambiguous final diagnosis could be established, the reference standard was set to ‘unclear.’ In such ‘unclear’ cases, the two imaging modalities were considered equal (no additional diagnostic value).

Demographic and clinical variables were then analyzed as potential predictors for perceived additional diagnostic value, including sex, side of neuropathy, presence of motor and sensory deficits, pain, prior surgery potentially related to neuropathy, electrophysiology results and nerve lesion characteristics (see below).

### Imaging

All patients underwent both HRUS and MRN of the upper extremity. Imaging was interpreted independently by two neuroradiologists (≥ 2 years of experience), with access to full clinical data. In case of disagreement, a third senior reader (≥ 5 years of experience) provided consensus.

HRUS was performed using an 18-MHz linear transducer (Siemens Acuson S2000) on the affected side, with contralateral assessment when indicated. Major nerves were systematically examined from the wrist to the supraclavicular plexus. HRUS typically preceded MRN on the same day. HRUS findings were retrospectively compared with MRN results and the final composite diagnosis.

MRN was acquired on 3-T scanners (Magnetom Skyra, Verio, or TIM-Trio; Siemens Healthineers) using a large-coverage protocol (Supplementary Table 2). The brachial plexus was imaged supine; distal nerve segments were scanned prone with the arm extended, using an 8- or 15-channel coil. The arm was positioned nearly parallel to the B₀ field (≤ 10° deviation) to reduce magic angle artifacts [20, 21].

MR image assessment included nerve signal changes (T2 hyperintensity), fascicular enlargement, discontinuity, and compressive or enhancing lesions. Muscle denervation (edema, atrophy) was evaluated in both modalities.

Lesion location was classified as spinal, supraclavicular, infraclavicular, or based on median, ulnar, or radial nerve involvement at the upper arm, elbow, forearm, or wrist. Lesion extent was defined as involvement of one region versus two or more. Distribution was categorized as proximal (plexus to axilla), distal (beyond axilla), or combined. Nerve involvement was defined as mononeuropathy or multi-nerve involvement.

### Statistical analysis

Demographic and clinical characteristics were summarized using mean ± standard deviation (SD) for continuous variables and frequencies (*n* or %) for categorical variables.

Associations between clinical or demographic variables and the presence of perceived additional diagnostic value were first explored using univariable Chi-squared tests. The full cohort (*n* = 800) served as the reference population to examine the distribution and calculate subgroup-specific proportions of patients in whom one modality showed additional diagnostic value.

To assess potential multicollinearity among predictor variables, pairwise intercorrelations were evaluated using Cramér’s V. To identify independent predictors of perceived additional diagnostic value, multivariable binary logistic regression analysis was applied on the full cohort with “MRN perceived additional diagnostic value” (yes/no) as the dependent variable. Predictor variables included patient symptoms (motor and sensory deficits, pain), prior nerve-related surgery, electrophysiologic findings, nerve involvement pattern (mono- vs. multi-nerve), lesion distribution (peripheral only, proximal only, or both), and anatomical lesion extent (one vs. multiple regions affected). Missing data were handled using Multiple Imputation by Chained Equations (MICE) [22] with 100 imputed datasets, and pooled using Rubin’s rule [23]. All predictor variables were prespecified based on clinical relevance and plausibility and were included in the model without additional data-driven selection. Odds ratios with 95% confidence intervals are presented.

In addition, a Classification and Regression Tree (CART) model was constructed to further explore and visualize key predictors of perceived additional diagnostic value of MRN compared to HRUS using the full cohort [24] based on the same predictor variables as for the binary logistic regression analysis. The binary outcome variable was “MRN perceived additional diagnostic value.” Predictor variables were categorized as factors. Missing predictor values were handled using surrogate splits as implemented in the CART algorithm [25] and in the *rpart* package in R [26]. Model training was performed using five-fold cross-validation to reduce overfitting and assess generalizability. Tree growth was restricted to parent nodes with at least 100 observations and terminal nodes with at least 50 observations. The final model was visualized as a decision tree.

Statistical analyses were performed using RStudio (Posit team, 2025). RStudio: Integrated Development Environment for R. Posit Software, PBC, Boston, MA. URL http://www.posit.co/). A *p*-value < 0.05 was considered statistically significant.

## Results

### Participant characteristics

From the original cohort of 800 patients with clinically suspected upper extremity neuropathy, diagnostic findings were concordant between MRN and HRUS in 479 cases (59.9%), providing equivalent diagnostic information. The remaining 321 cases (40.1%) showed discordant findings between modalities. After excluding non-true positives, 275 patients (34.4%) met criteria adding diagnostic value and remained for analysis (Fig. 1). Among these, MRN provided perceived additional diagnostic value in 261 cases (94.9%), including 136 cases (49.5%) with negative HRUS but positive MRN findings, and 125 cases (45.5%) where both modalities were positive but MRN provided additional clinically relevant diagnostic information (examples shown in Supplementary Figs. 1 and 2). In contrast, HRUS provided additional diagnostic value in only 14 cases (5.1%), all of which also showed positive MRN findings, but with HRUS offering additional or clearer lesion characterization (for details, see Supplementary Table 1).

Among patients in whom MRN provided perceived additional diagnostic value was found (*n* = 261), motor deficits were present in 77.0%, sensory deficits in 55.6%, and pain in 39.1%. Electrophysiological studies were available in 57.5% of these patients, with 76.7% showing abnormal findings. Lesions involved multiple nerves in 55.6% of cases, with proximal nerve involvement in 58.6% (including 31.4% with isolated proximal-only lesions). Lesions extended across more than one anatomical region in 59.4% of cases. These characteristics are detailed in Table 1, alongside the corresponding data for the small HRUS additional diagnostic value subgroup and the larger group where no additional diagnostic value was found.

**Table 1:** Descriptive comparison of clinical and lesion characteristics in cases where either high-resolution ultrasound (HRUS), MR neurography (MRN), or no modality provided perceived additional diagnostic value

Clinical and lesion characteristics	Category	Additional diagnostic value		No additional diagnostic value (*n* = 525)
		HRUS (*n* = 14)	MRN (*n* = 261)	
Age ± SD	Years	42.8 ± 16.9	49.8 ± 16.4	47.7 ± 16.5
Sex	Male	57.1%	54.8%	53.7%
	Female	42.9%	45.2%	46.3%
Affected side	Left	57.1%	49.4%	44.6%
	Right	42.9%	50.6%	55.4%
Prior surgery	No	7.1%	64.0%	57.3%
	Yes	92.9%	36.0%	42.7%
Electrophysiological studies	Normal	7.1%	23.3%	59.0%
	Abnormal	50.0%	76.7%	41.0%
Motor deficits	Absent	21.4%	22.2%	37.3%
	Present	78.6%	77.0%	62.7%
Sensory deficits	Absent	14.3%	31.4%	27.2%
	Present	64.3%	55.6%	65.0%
Pain	Absent	35.7%	47.1%	40.6%
	Present	35.7%	39.1%	51.4%
Lesion location	Proximal only	0%	31.4%	6.9%
	Peripheral only	100%	40.6%	61.1%
	Combined	0%	27.2%	4.4%
Nerve involvement	Mononeuropathy	85.7%	43.7%	69.1%
	Multi-nerve involvement	14.3%	55.6%	18.3%
Lesion extent	One anatomical region	42.9%	39.8%	49.1%
	Two or more regions	57.1%	59.4%	23.4%

Percentages of each characteristic are shown for cases in which each modality perceived additional diagnostic value

### Univariable associations with MRN perceived additional diagnostic value

Univariable analysis across the full cohort (*n* = 800) revealed significant associations between diagnostic value added by MRN and several clinical factors. Proximal nerve involvement was strongly associated with MRN perceived additional diagnostic value (69.5% for proximal-only and 75.5% for combined lesion patterns vs. 23.9% for peripheral-only; *p* < 0.001). Similarly, involvement of more than one nerve was frequently observed among cases with additional diagnostic value (59.4% for multi-nerve involvement vs. 23.3% for mononeuropathy; *p* < 0.001), and cases with lesion extent of more than one anatomical region showed higher rates of MRN additional diagnostic value (54.0% vs. 28.3%; *p* < 0.001). Motor deficits were also more frequently observed in patients with diagnostic value added by MRN (37.2% in those with deficits of any severity vs. 22.6% without deficits; *p* < 0.001). Furthermore, additional diagnostic value of MRN was more common in patients without pain (35.9% vs. 27.1% with pain; *p* = 0.01) and slightly more frequent in those without sensory deficits (36.0% vs. 29.2%; *p* = 0.08), although the latter did not reach statistical significance. A modest association was also observed for prior surgery, with diagnostic value added by MRN more likely in patients without a history of surgical intervention related to the neuropathy (35.8% vs. 28.2%; *p* = 0.03). These results are summarized in Table 2.

**Table 2:** Univariable analysis of predictors for perceived additional diagnostic value of MR neurography (MRN) compared to high-resolution ultrasound

Predictor variable	Category	Additional diagnostic value by MRN within each category (%) (*n*/*N*)	*p*-value
Sex	Male	33.2% (143/431)	0.78
	Female	32.0% (118/369)	
Affected side	Left	34.7% (129/372)	0.28
	Right	30.8% (132/428)	
Lesion location	**Proximal only**	**69.5% (82/118)**	**< 0.001**
	**Combined proximal + peripheral**	**75.5% (71/94)**	
	Peripheral only	23.9% (106/443)	
Nerve involvement	Mononeuropathy	23.3% (114/490)	**< 0.001**
	**Multi-nerve involvement**	**59.4% (145/244)**	
Lesion extent	One anatomical region	28.3% (104/368)	**< 0.001**
	**Two or more regions**	**54.0% (155/287)**	
Motor deficits	Absent	22.6% (58/257)	**< 0.001**
	**Present**	**37.2% (201/377)**	
Pain	**Absent**	**35.9% (123/343)**	**0.01**
	Present	27.1% (102/377)	
Sensory deficits	Absent	36.0% (82/228)	0.08
	Present	29.2% (145/496)	
Prior surgery	**No**	**35.8% (167/467)**	**0.03**
	Yes	28.2% (94/333)	

Percentages indicate the proportion of patients within each category who demonstrated perceived additional diagnostic value by MRN, with absolute numbers shown. *p*-values are derived from Chi-squared tests comparing the distribution of perceived additional diagnostic value by MRN across categories. Statistically significant categories (*p* < 0.05) associated with perceived additional diagnostic value by MRN are bolded

In contrast, HRUS added diagnostic value compared to MRN in only 14 cases, primarily due to artifacts from osteosynthetic material (For details, see Supplementary Table 1). A meaningful formal statistical analysis to determine individual predictors could not be carried out.

### Multivariable modeling of MRN perceived additional diagnostic value

Associations among predictor variables were generally weak to moderate (all Cramér’s V < 0.48), with the exception of nerve involvement (mononeuropathy/multi-nerve involvement) and lesion location (proximal/peripheral/combined), which showed a partial overlap (Cramér’s V = 0.61).

**Fig. 2:**
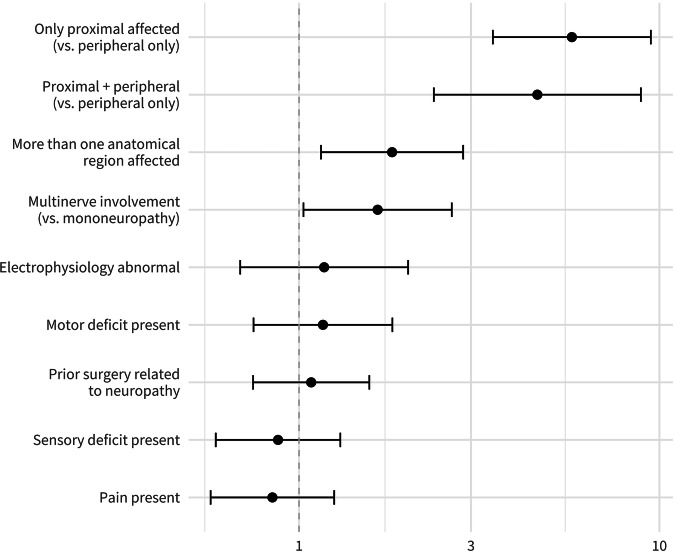
Multivariable logistic regression analysis of predictors for MRN perceived additional diagnostic value. This forest plot displays the adjusted odds ratios with 95% confidence intervals from a multivariable logistic regression model. Missing data were handled using Multiple Imputation by Chained Equations (MICE) with 100 imputations, and pooled estimates were calculated using Rubin’s rules

Multivariable logistic regression analysis identified lesion location as the strongest independent predictor of perceived additional diagnostic value of MRN. Compared to peripheral-only lesions, proximal-only involvement was associated with substantially higher odds of diagnostic value added by MRN (OR 5.72, 95% CI: 3.42–9.56), as were lesions involving both proximal and peripheral segments (OR 4.59, 95% CI: 2.31–9.09). Lesion extent affecting more than one anatomical region also predicted MRN additional diagnostic value (OR 1.81, 95% CI: 1.14–2.87), as did multi-nerve involvement (OR 1.65, 95% CI: 1.03–2.63) (Fig. 2).

**Fig. 3:**
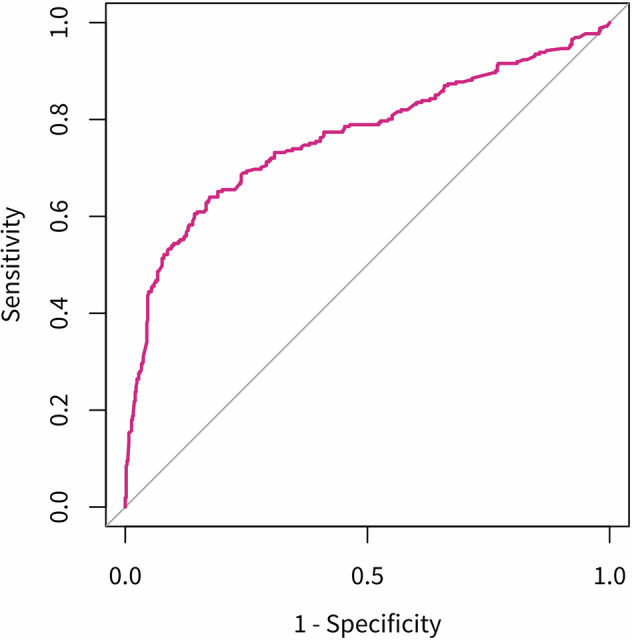
Receiver operating characteristic (ROC) curve for predicting perceived additional diagnostic value of MR neurography (MRN). The curve shows the performance of the final multivariable logistic regression model predicting perceived additional diagnostic value of MRN. Predicted probabilities were pooled by averaging across 100 multiply imputed datasets, and the area under the curve (AUC) was 0.76

Other clinical features—including motor deficit, sensory deficit, pain, prior surgery, and abnormal electrophysiological findings—were not significantly associated with diagnostic value added by MRN in the final model (Fig. 2). The model achieved a pooled area under the receiver operating characteristic (ROC) curve (AUC) of 0.76 (Fig. 3).

**Fig. 4:**
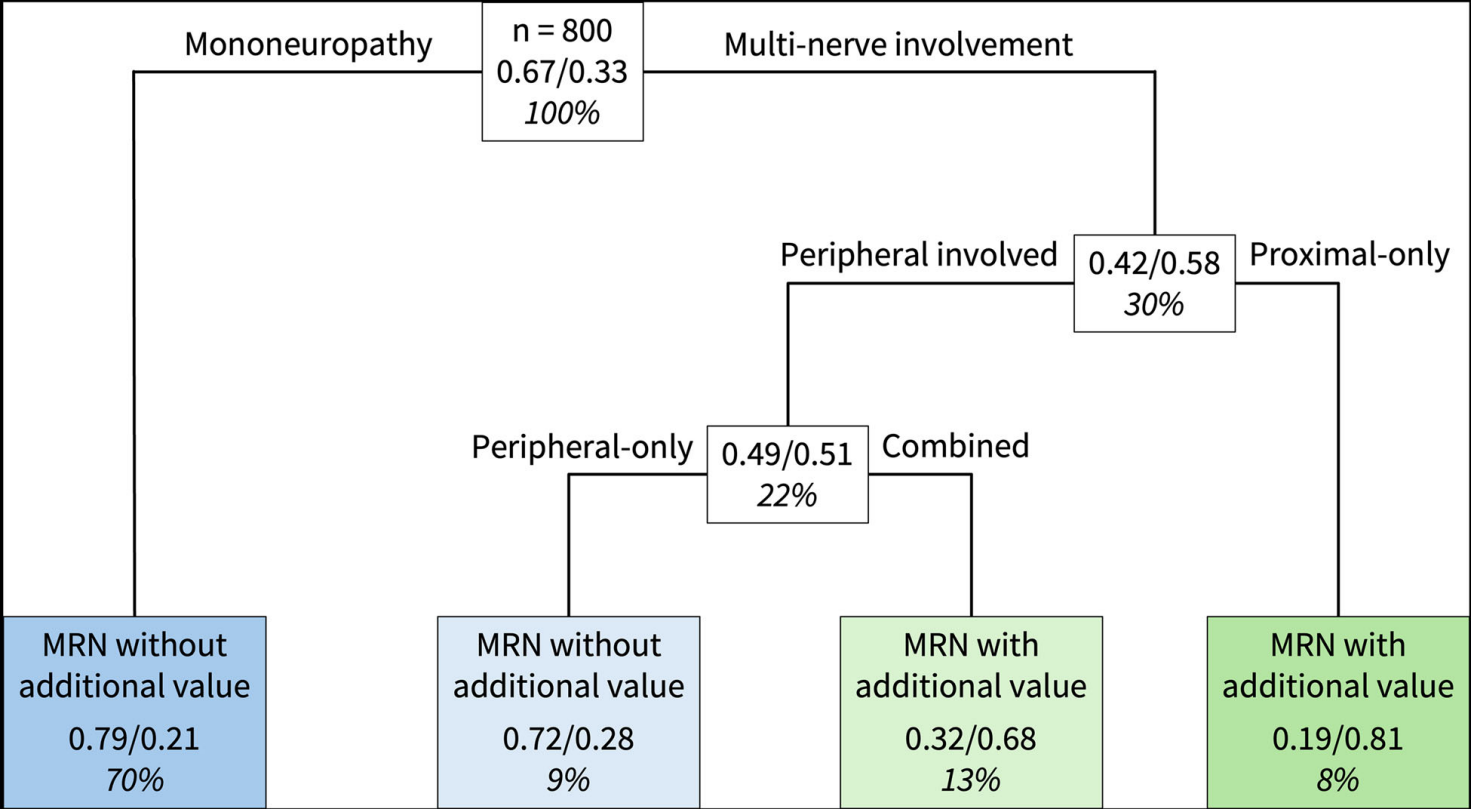
Classification and regression tree (CART) predicting MRN perceived additional diagnostic value. Each node displays the predicted class probabilities (MRN without perceived additional diagnostic value/MRN with perceived additional diagnostic value) and the percentage of the total study population represented in that node. Terminal nodes represent final classifications based on majority class

In addition, a classification and regression tree (CART) model was used to identify and visualize key predictors of MRN perceived additional diagnostic value. The final classification tree included eight predictor variables and produced three internal splits. The root node comprised all 800 patients, including 539 without and 261 with MRN perceived additional diagnostic value. The first split was based on nerve involvement pattern, separating patients with mononeuropathy from those with multi-nerve involvement. Subsequent splits occurred within the multi-nerve group based on lesion distribution (proximal, peripheral, or combined), followed by additional stratifications by lesion location. These splits yielded terminal nodes with distinct class distributions and probabilities (Fig. 4).

The final model achieved a mean cross-validated classification accuracy of 76.1%. The complexity parameter (cp) was 0.0587.

## Discussion

In the original prospective observational study of 800 patients with suspected upper extremity peripheral neuropathies, MRN achieved higher accuracy and sensitivity, whereas HRUS showed higher specificity [17]. Building on these findings, the present subanalysis focused specifically on cases with discordant or incremental findings between MRN and HRUS to explore clinical and anatomical predictors of perceived additional diagnostic value in routine clinical practice, defined as a modality’s ability to provide diagnostic confidence, lesion detection, or characterization that directly influences patient management. Unlike previous work focusing primarily on accuracy metrics such as sensitivity and specificity [15, 27], this exploratory subanalysis emphasizes clinical utility and practice-patterning rather than general diagnostic superiority or objective diagnostic accuracy outcome. Prior studies have underscored the strengths of MRN in visualizing deep and complex anatomy, such as the brachial plexus and detecting subtle pathological changes not visible on HRUS [13, 28]. However, these investigations did not systematically assess clinical or anatomical predictors of additional diagnostic contribution in direct comparison between the two modalities.

Defining a correct reference standard was a key challenge at the outset: In peripheral nerve neuropathies, diagnoses typically emerge from the combined interpretation of clinical examination and electrodiagnostic testing, often with imaging playing a decisive role in localizing pathology or clarifying etiology. For this reason, we used a composite clinical reference reflecting routine practice and explicitly framed our objective, including imaging results within that clinical workflow, which we think represents the most suitable approach to approximate “the ground truth” in the diagnostic challenge of peripheral neuropathies in a real-world scenario.

MRN showed additional diagnostic value in approximately one-third of the study cohort, underscoring its clinical relevance. HRUS provided additional diagnostic value in less than 2% of the total cohort, usually when MRN was degraded by artifacts from metal implants. This small but notable subset shows that HRUS can provide diagnostic value when MRN is technically limited or inconclusive. At the same time, our findings emphasize the perceived additional diagnostic value of MRN in defined clinical scenarios and support its targeted use in specific diagnostic workflows.

Topographical lesion characteristics—such as proximal location and multi-nerve involvement—emerged as the most robust predictors of MRN perceived additional diagnostic value in routine clinical practice, supporting anatomical plausibility and prior observations [29]. Consistent with its superficial imaging capabilities, HRUS provided additional diagnostic value only in a minority of cases, particularly when MRN was impaired by metal artifacts, as reported in earlier studies [30, 31].

To our knowledge, this is the first study to employ a classification tree model in guiding imaging modality selection in peripheral neuropathies for routine clinical practice. Although this approach may be biased by institutional decision behavior, it may aid clinicians in balancing cost-effectiveness, availability, and diagnostic performance—a challenge highlighted in recent imaging pathway studies [32]. Of particular interest is the role of multi-nerve involvement as the first decision node in the classification tree. Although partially collinear with lesion distribution (proximal/peripheral/combined), both variables were retained due to their different clinical implications. Multi-nerve involvement often indicates widespread disease (e.g., inflammatory or systemic), while proximal vs. distal location aids localization and differential diagnosis [33, 34]. Despite some overlap, their combination improved stratification and mirrored clinical diagnostic reasoning.

Motor deficits were associated with additional diagnostic value for MRN in univariable analysis, but not in multivariable modeling. This may reflect overlap with topographical factors, as motor impairment can occur in both proximal and distal lesions and can be associated with greater lesion extent or multi-nerve involvement [29, 35]. Once these anatomical and distributional factors were accounted for, motor deficits may not have provided additional independent predictive value. Pain and sensory symptoms, though clinically relevant, were less predictive of imaging yield. This likely stems from their subjective nature and overlap with non-neuropathic causes like musculoskeletal or functional pain [36]. These findings support emphasizing objective, lesion-based variables over symptom-based indicators in selecting the most appropriate imaging modality.

Our results suggest a more tailored imaging strategy for peripheral neuropathy clinical assessment, although the endpoint employed in our exploratory analysis comes with important limitations and weaknesses (see below) and may not be interpreted as an objective diagnostic accuracy outcome. Rather than defaulting to MRN or HRUS across all cases, clinicians can integrate lesion characteristics and clinical context to guide imaging. MRN should be favored when proximal or multi-nerve involvement is suspected. HRUS remains valuable as a low-cost, widely available tool, especially for superficial, focal, and distal lesions, or when MRN is contraindicated or compromised. In addition, HRUS allows dynamic assessment of periarticular nerve segments in different angles and during movement, which is particularly useful in specific entrapment syndromes.

This study comes with a number of important limitations. First, it was conducted as a single-center study in a tertiary referral center with access to advanced imaging and subspecialist interpretation, with institution-specific results that were not externally validated, which may limit generalizability. Second, the endpoint of perceived additional diagnostic value, while clinically grounded, is based on a non-algorithmic, clinician-adjudicated and therefore inherently subjective composite reference standard, potentially including qualitative judgements based on radiologists’ perceptions despite a set of predefined criteria and independent reading. Third, including both imaging modalities within the composite reference standard may introduce an incorporation bias, thus limiting the ability to make claims about intrinsic diagnostic accuracy. Moreover, a composite reference standard should ideally engage a unified transparent decision rule for diagnostic decision-making to optimize reproducibility and to minimize the risk of an additional verification bias. Therefore, the endpoint variable *perceived additional diagnostic value* needs to be interpreted with caution due to its potentially subjective and institution-specific, not externally validated nature. As such, it may not be interpreted as an objective diagnostic accuracy outcome. Fourth, the small number of cases with HRUS additional diagnostic value restricted our ability to model predictors for this subgroup. Also, HRUS is inherently operator dependent, and diagnostic performance can vary with experience, equipment, and protocol adherence, so that the generalizability of our findings within a tertiary referral center to other practice environments may be limited. Next, the study design did not account for cost-effectiveness considerations, external validation or patient preferences, which may influence real-world imaging decisions. Finally, the limited availability of MRN and typically longer waiting times compared to HRUS may further affect its practical integration into clinical pathways.

In conclusion, MRN was found to provide perceived additional diagnostic value in routine clinical practice compared to HRUS in a clinically relevant proportion of patients with upper extremity neuropathies, particularly those with proximal, multi-nerve, or extensive lesions. These exploratory results support an individualized imaging approach with MRN to be favored in complex or ambiguous clinical scenarios, and HRUS as a complementary or alternative tool when MRN is unavailable, contraindicated, or metal artifact-prone.

## Supplementary information

Supplementary information

## Abbreviations

HRUS: High-resolution nerve ultrasound
MRN: Magnetic resonance neurography
