# Supplementary material for: High-resolution ultrasound vs. MR neurography in upper extremity neuropathies: exploratory analysis of perceived additional diagnostic value in routine clinical practice
Source: Eur Radiol. 2026 Apr 23;36(9):7010–9. doi: 10.1007/s00330-026-12545-0 (PMC13451437; doi:10.1007/s00330-026-12545-0)

High-Resolution Ultrasound vs. MR Neurography in Upper Extremity Neuropathies: Exploratory Analysis of perceived Additional Diagnostic Value in Routine Clinical Practice

ELECTRONIC SUPPLEMENTARY MATERIAL

Supplementary Table 1 Criteria for perceived additional diagnostic value based on diagnostic information. Definition and case counts for each criterion used when both MRN and HRUS showed positive findings, but one modality added clinically crucial information.

Criterion	Description	Case count	
		**MRN**	**HRUS**
**A. Overcoming Limitations of the other modality**			
A.1 Metal artifact	The modality that additional diagnostic value enabled clear diagnostic evaluation in a region compromised by metal-induced artifacts in the other modality.	-	10
A.2 Anatomical/technical limitation	Successful visualization of a lesion that was not imaged by the other modality due to anatomical location or technical constraints, or because it lay outside the scan range typically covered by that modality.	20	-
**B. Detection of additional pathology**			
B.1 Additional level detected	At least one additional affected anatomical level was visualized, whether symptomatic, asymptomatic, or already suspected clinically.	51	-
B.2 Additional nerve detected	At least one additional affected nerve was visualized, regardless of clinical symptoms or prior electrophysiological findings.	36	-
B.3 Muscle denervation	Denervation patterns visible on one modality supported diagnosis or timing; relevant for clinical or forensic interpretation.	5	-
B.4 Relevant incidental finding	One modality revealed additional clinically or diagnostically important incidental findings.	5	-
**C. Contribution to diagnostic reasoning**			
C.1 Suggestive diagnosis	One modality allowed formulation of a specific suspected diagnosis, while the other provided only descriptive findings.	51	-
Narrowed differential diagnoses	One modality enabled (or excluded) focused differential diagnoses.	2	-

D. Diagnostic value beyond visualization			
D.1 Better contrast/resolution	Lesion was better distinguishable from surrounding structures or more clearly related to adjacent anatomy in one modality.	10	1
D.2 Higher diagnostic confidence	One modality allowed a more confident interpretation (due to clearer contrast or resolution, precisely delineated nerve damage).	7	3
D.3 Confirmed correct localization	Only one modality enabled final confirmation of correct lesion location.	3	-

Supplementary Table 2 : Overview of MR Neurography protocol parameters. TR = repetition time; TE = echo time; STIR = short tau inversion recovery; SPAIR = spectral attenuated inversion recov-ery; fs = fat-saturated; CE = contrast-enhanced; TRUFI = true fast imaging with steady-state precession; cor = coronal; sag = sagittal; axial = axial plane.

Sequence	TR (ms)	TE (ms)	Matrix	Slice thickness (mm)	Pixel spacing (mm)	Acquisition time (min)
Brachial Plexus						
T2-w SPACE STIR cor	3800	267	320 × 314	0.8	0.78 × 0.78	07:45
T2w SPAIR sag-oblique	5530	45	320 × 198	3.0	0.47 × 0.47	07:46
Arm and Wrist						
T2w fs axial	7020	52	512 × 358	3.0	0.25 × 0.25	07:17
T1w axial	848	20	512 × 230	3.0	0.33 × 0.33	06:00
T1w fs axial + CE	969	20	512 × 230	3.0	0.33 × 0.33	06:48
TRUFI cor	8.55	3.74	384 × 365	0.4	0.40 × 0.40	06:07

Supplementary Figure 1: Case example perceived additional diagnostic value by MRN based on criterion C.1 (“Suggestive diagnosis”) as defined in Supplementary Table 1. A 73-year-old male patient presenting with thenar atrophy, recurrent pain and paraesthesia in the median nerve distribution of the left hand after three carpal tunnel surgeries (last surgery 3 years ago). Excessive scarring and a painful palpable mass at the palmar wrist were present on clinical examination. On imaging, the median nerve (dotted circle) was anteriorly displaced and severely flattened by a circumscribed, septated, 1.7 cm × 2.0 cm mass (white arrows) with mainly hyperechoic signal on nerve HRUS (A), heterogenous T1- and T2-hyperintensity (B-D), no contrast-enhancement and thin connection to the radioscaphoid and scaphotrapezoid joint, consistent with a ganglion cyst. Discontinuity and neuroma formation of the median nerve were ruled out by both imaging modalities. The patient underwent nerve decompression and histology proved the suspected diagnosis of a ganglion cyst. Abbr.: HRUS = high-resolution ultrasound; TRUFI = true fast imaging with steady-state free precession sequence, T2fs = fat-saturated T2 sequence.

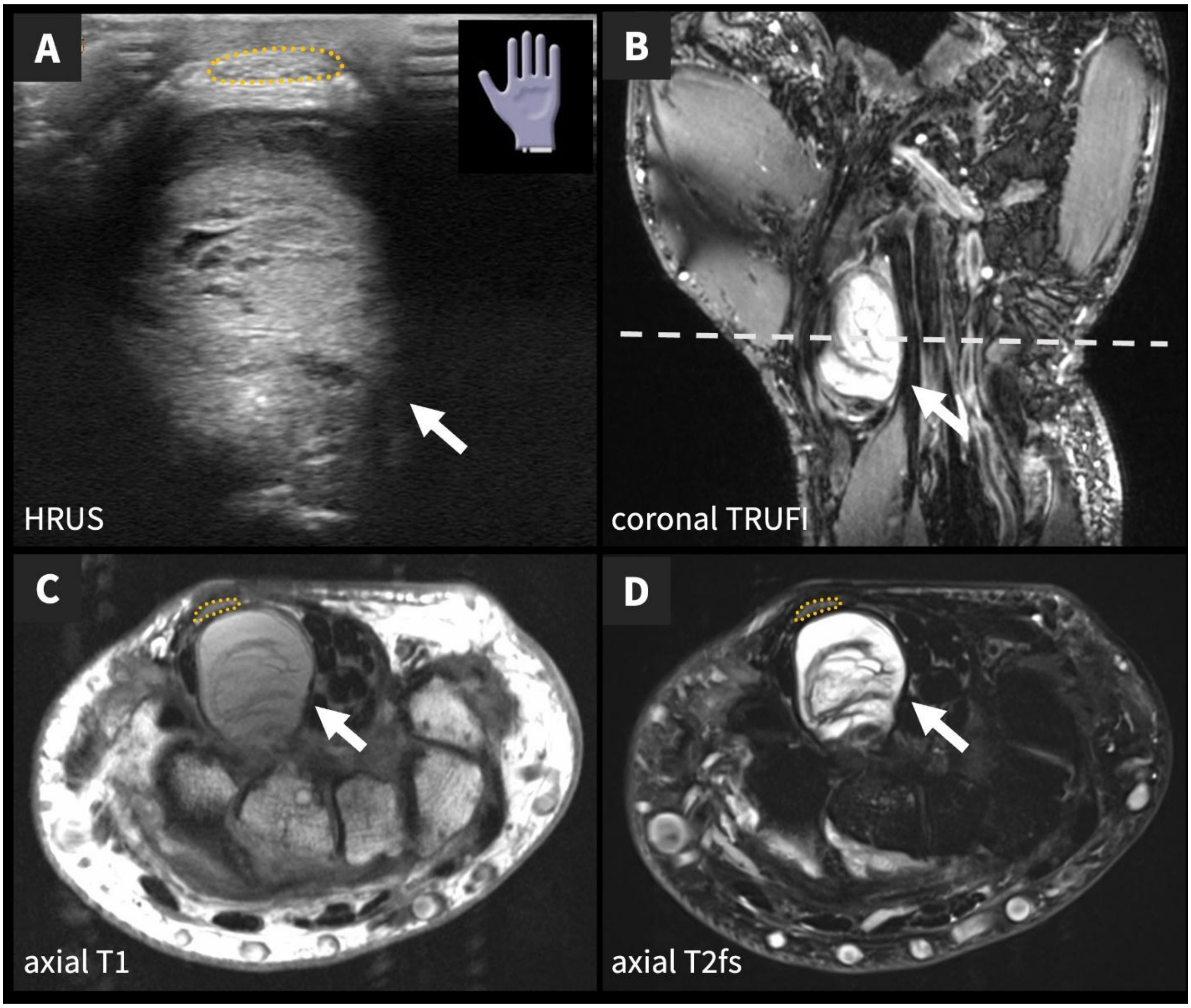

Supplementary Figure 2: Case example of perceived additional diagnostic value by MRN based on criterion B.1 (“Additional level detected”) and C.1 (“Suggestive diagnosis”) as defined in Supplementary Table 1. A 67-year-old female patient presenting with slowly progressive *pain radiating from the right shoulder into the ipsilateral arm.* She had a history of recurrent right-sided breast cancer six years ago treated with mastectomy, axillary lymphadenectomy and adjuvant radiochemotherapy. (A) Nerve ultrasound showed a heterogenous, 2.8 × 1.7 cm mass with ill-defined margins in the course of the infraclavicular right brachial plexus, however, failed to visualise the exact relation to the nerve plexus. MR neurography revealed direct contact of the T2-hyperintense (B), strongly enhancing (E) mass (*) to the lateral fascicle (dotted circles in B, D, E) and detected two additional lesions invisible on ultrasound at supraclavicular height without direct contact to the trunci (C) and retroclavicular with direct proximity to the lateral fascicle (D). MRN report suspected malignant lymph nodes with plexus infiltration (arrows in B, D-F) at two sites, proven to be breast cancer metastases on histology. Abbr.: cl = clavicle; cor. = coronal; sag. = sagittal; T2fs = fat-saturated T2 sequence; T1fs CE = fat-saturated contrast-enhanced T1 sequence.

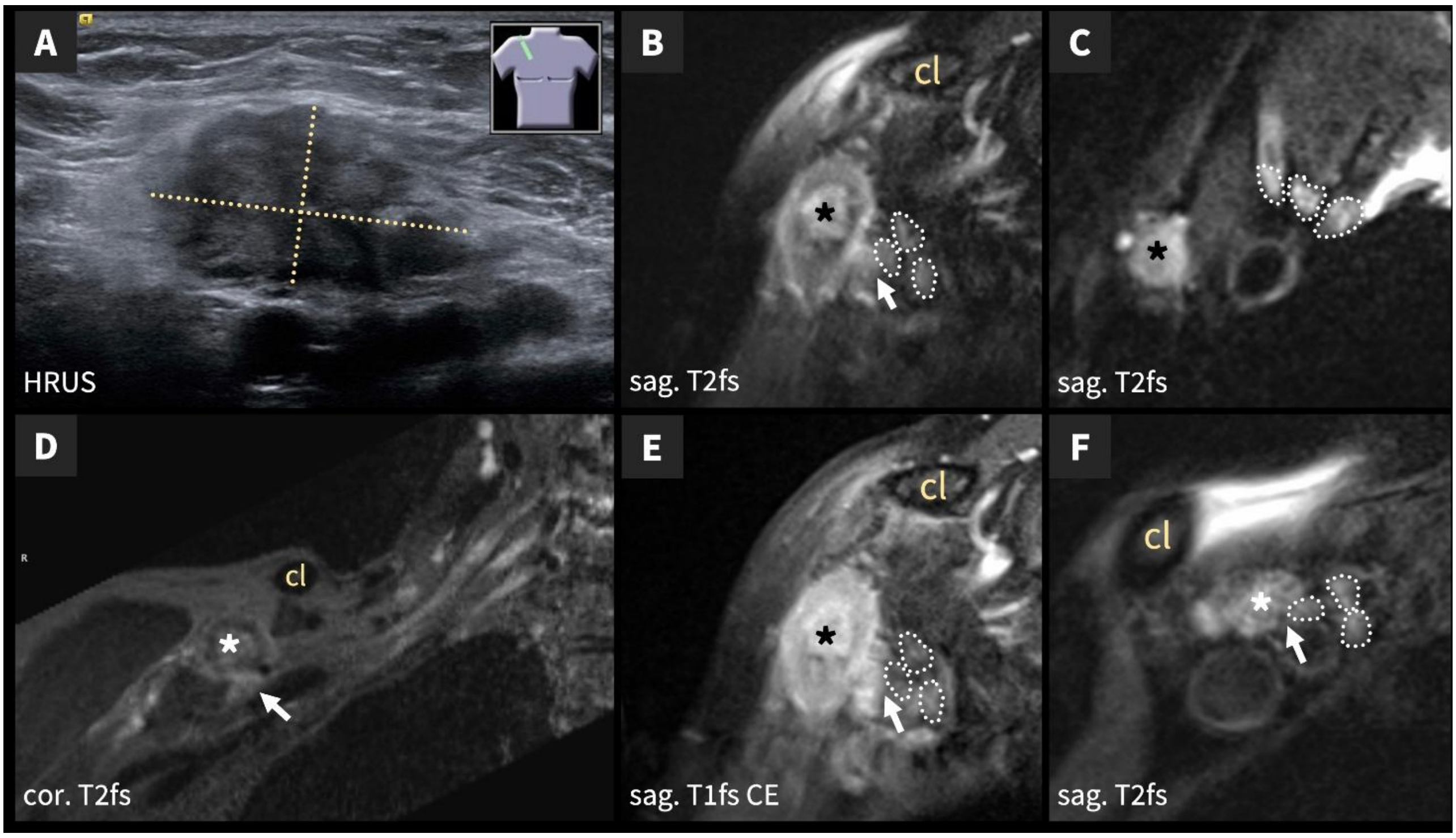

Supplement: Supplementary file 1 [file 330_2026_12545_MOESM1_ESM.pdf]
